# Editorial: Promoting mental health among healthcare professionals

**DOI:** 10.3389/fpsyt.2026.1985585

**Published:** 2026-09-29

**Authors:** Mingzhou Gao, Yelong Shen, Xia Li, Hao Zhang

**Affiliations:** 1 Innovation Research Institute of Chinese Medicine and Pharmacy, Shandong University of Traditional Chinese Medicine, Jinan, Shandong, China; 2 Shandong Provincial Hospital Affiliated to Shandong First Medical University, Jinan, Shandong, China; 3 Key Laboratory of Traditional Chinese Medicine Classical Theory, Ministry of Education/Shandong Key Laboratory of Innovation and Application Research in Basic Theory of Traditional Chinese Medicine/Shandong Provincial Engineering Research Center for the Prevention and Treatment of Major Brain Diseases with Traditional Chinese Medicine, Shandong University of Traditional Chinese Medicine, Jinan, Shandong, China

**Keywords:** anxiety, burnout, depression, healthcare professionals, mental health

## Introduction

Mental health among healthcare professionals has emerged as one of the most pressing occupational-health challenges of our era (1). Long before the COVID-19 pandemic, sustained occupational strain, heavy workloads and the emotional toll of caring for vulnerable patients rendered healthcare workers at elevated risk of burnout, compassion fatigue, anxiety and depression. The pandemic dramatically exacerbated these stressors, and the psychological sequelae have persisted long afterwards (2).

This Research Topic compiles 33 articles, comprising original empirical investigations, cross-sectional and longitudinal surveys, group concept-mapping analyses, integrative reviews and systematic reviews. Collectively, these contributions advance scholarly understanding of mental health in the healthcare workforce by illuminating pathogenic mechanisms, protective psychological resources, context-specific vulnerabilities and implementable intervention pathways. Characterized by geographical and methodological diversity, the studies identify universal challenges while offering context-tailored solutions, generating a comprehensive evidence base for policymakers, healthcare administrators and clinicians striving to build a sustainable, mentally healthy workforce.

## The burden of mental health challenges

Several contributions provide critical epidemiological evidence on the scope of mental health challenges facing healthcare workers. A landmark systematic review and meta-analysis (Frodsham et al.) documented that the pooled prevalence of probable PTSD among healthcare workers was 15.5% prior to the pandemic, rising significantly to 24.8% during COVID-19, peaking in 2020 before returning to pre-pandemic levels in 2022. And school nurses in California during the pandemic (Best et al.) reported 40% burnout prevalence, with burnout severity significantly correlated with higher moral distress scores, driven largely by systemic barriers including inability to provide preventive care. Turkish nurses (Yildrim et al.) experienced moderate COVID-19 phobia, with 46.8% languishing in the emotional subdimension, and gender, education, perceived health status, and intention to quit predicting 17% of phobia variance.

In Sub-Saharan Africa (Khangamwa-Singano et al.), burnout prevalence rates were reported as high as 54.5% in Tanzania and 68% in Malawi, underscoring how resource constraints and chronic staff shortages amplify vulnerability in low-resource settings. In conflict-afflicted areas, a study among Palestinian healthcare professionals (Nabulsi et al.) found anxiety prevalence of 51.5% and depression of 45.3%, revealing the compounding effects of war and political violence on mental health. Similarly, Ecuadorian medical residents (Sanchez et al.) showed substantial burdens with 38.8% depression, 33.9% anxiety, and 12.1% hazardous drinking prevalence. Among Chinese residency trainees (Wei et al.), occupational burnout prevalence reached 70.48%, with 68.37% reporting at least one adverse practice experience—verbal abuse (51.51%), personal services demand (49.25%), and gender discrimination (47.14%) being the most common. Physical and emotional abuse exposures showed the strongest associations with burnout scores. Acupuncture practitioners in China’s public hospitals (Dong et al.) demonstrated moderate burnout levels, with emotional exhaustion, depersonalization, and reduced personal accomplishment as primary dimensions, influenced by individual, organizational, occupational, and societal factors.

Beyond these broad estimates, specific occupational contexts reveal distinctive patterns of risk. Intensive care unit professionals face compassion fatigue characterized by emotional exhaustion and reduced empathic capacity (Olander et al.). Psychiatric nursing involves high levels of emotional labor with dual pathways—surface acting (maladaptive) versus deep acting (adaptive)—with surface acting emerging as the primary bridge linking emotional labor to burnout (Huang et al.). The mediating role of emotion regulation, particularly cognitive reappraisal, was confirmed in the relationship between indecent work perception and burnout among psychiatric nurses, accounting for approximately 17% of the total effect (Tang et al.). Furthermore, occupational groups within emerging healthcare service models also exhibit distinctive risk profiles. A cross-sectional study focusing on nurses delivering “Internet+” home care services (Wang et al.) revealed that this group reported low job burnout scores. But positive coping styles were negatively correlated with total burnout scores, whereas negative coping styles were positively correlated with total scores. These findings suggest that as healthcare service models undergo digital transformation, attitudes toward coping with burnout may become key to breaking the deadlock.

## Protective factors and mechanisms

A substantial cluster of studies investigated the protective factors that buffer healthcare workers against mental health deterioration. Social support and psychological resilience emerged as critical protective resources. One study (Li et al.) demonstrated that social support and psychological resilience significantly impact comprehensive wellbeing, with psychological resilience serving as a key mediator that enhances the positive effects of social support on overall wellbeing. In a related investigation (Li et al.) examining township healthcare workers in Lanzhou, sleep disorders were detected in 55.70% of participants, with social support playing a partial mediating role in the relationship between sleep quality and mental health (indirect effect proportion: 7.36%). These findings underscore the interconnectedness of sleep, social connectedness, and psychological resilience as interdependent pillars of mental health. A study of nurses in Chengdu (Hou et al.) found that 35.7% reported anxiety, with psychological resilience partially mediating the relationship between social support and anxiety (accounting for 21% of the total effect); the pathway mediated by resilience was strongest for subjective support, with indirect effect constituting 41% of its total impact.

Besides, the distinction between mentally active versus passive sedentary behaviors was explored among Chinese nurses (He et al.). Nurses engaging in mentally active sedentary behaviors—such as working, reading, hobbies, transportation, and chatting—for more than 4 hours/day had 47% lower odds of burnout compared to those with less than 1 hour/day. This inverse association remained after excluding transport-related sedentary behavior, while mentally passive sedentary behavior showed no significant association—a distinction offering practical, actionable guidance for workplace wellness initiatives.

During crisis contexts, resilience and sense of agency were examined among Israeli healthcare professionals during mass casualty incidents and violent conflict (Link et al.). Psychological capital and resilience were strongly associated with perceived agency, surpassing prior experience as predictors. Paramedics/EMTs reported higher agency than nurses/physicians, and spirituality also predicted agency. Empathy, a critical capacity in medical education, was found to have dual-pathway associations with mental health among medical students (Zhang et al.). Empathy was positively associated with psychological distress, more rumination thinking, and better interpersonal competence. Interpersonal competence and rumination fully mediated the empathy-distress relationship—rumination linked to higher distress, while interpersonal competence was associated with reduced distress. The dynamic model of mental health proposed by one contribution (Zhang and Zhang) integrates stress, coping mechanisms, and resilience, incorporating individual, organizational, and socio-cultural factors, while emphasizing the importance of addressing moral dilemmas, high workloads, and systemic deficiencies.

## Innovative interventions and support strategies

Several articles advanced the evidence base for interventions. A systematic review of biofeedback interventions for healthcare professionals (Cantone et al.) found consistent improvements in perceived stress, anxiety, depressive symptoms, emotional regulation, and resilience. Heart rate variability biofeedback and respiratory sinus arrhythmia training demonstrated the strongest effects, with physiological benefits including increased HRV, decreased sympathetic arousal, and improved autonomic balance. Despite methodological limitations including small sample sizes and heterogeneity, biofeedback was established as a feasible and potentially effective strategy for mitigating occupational stress.

Digital mental health support needs were systematically analyzed using the Kano model among 1,571 Chinese nurses (Chen et al.). Of 26 identified needs, 8 were classified as must-be attributes, 8 as one-dimensional attributes, 7 as attractive attributes, and 3 as indifferent attributes. This nuanced framework provides a roadmap for designing digital mental health services that align with what nurses truly value and require. A cluster randomized controlled trial evaluated an integrated empowerment and narrative nursing program among 172 neonatal nurses (Liu et al.). The I-ENP group demonstrated significantly greater improvement in professional identity, with emotional exhaustion and depersonalization declining significantly and personal accomplishment increasing. These benefits were largely sustained at 9 months, and turnover intention was significantly lower in the I-ENP group.

A psychological program promoting posttraumatic growth was developed and validated among nursing trainees (Sim et al.), consisting of eight sessions addressing physical and emotional recognition, meaning-making, PTG reflection, happiness and value promotion, and hope inspiration. The treatment group showed increased PTG, resilience, mental wellbeing, and emotion processing post-intervention, maintained at follow-up, with a trend toward decreased burnout. Mental health literacy assessment was advanced through the validation of the Polish version of the Mental Health Literacy Scale among nursing students (Nowicki et al.). The 33-item MHLS-PL demonstrated good reliability, with independent predictors including age, education level, and interaction with persons diagnosed with mental disorders.

## Organizational and systemic approaches

Beyond individual-level interventions, several articles addressed organizational and systemic strategies. The integrative review on burnout management in Sub-Saharan Africa (Khangamwa-Singano et al.) synthesized evidence across Uganda, Mozambique, Botswana, and South Africa, identifying adaptive coping strategies—supportive supervision, workplace wellness activities, peer support, and culturally embedded coping practices—that enhanced motivation, job satisfaction, and emotional resilience. While self-distraction (traditionally labelled maladaptive) may be associated with lower burnout levels in the short term, prolonged use may mask underlying stressors and increase vulnerability to chronic burnout.The group concept mapping study with ICU professionals (Olander et al.) generated five clusters of coping strategies: (1) meaning making through a person-oriented perspective; (2) supportive professional practice; (3) peer support and empathic teamwork; (4) work-life balance; and (5) personal responsibility. Peer support was rated highest in terms of use, while supportive professional practice was rated highest in importance but low in use—indicating an implementation gap that requires organizational attention.

Work-family conflict among healthcare worker fathers during COVID-19 (Mogwitz et al.) was assessed across 2,844 fathers at four time points from 2020 to 2022. Fathers reported higher work-family conflict comparable to mothers, with risk factors including higher workload, exhaustion, moral concerns, and lower institutional trust. Exploratory longitudinal evidence pointed to a growing protective role of institutional trust against work-family conflict over time, underscoring that work-family conflict among HCW fathers is a structural issue requiring targeted workplace adaptations. Work-related wellbeing among hepatobiliary surgical nurses (Wang et al.) was shaped by both occupational stress and internal psychological resources. Structural equation modeling demonstrated good fit, indicating that resilience and professional benefits had direct positive effects on wellbeing, occupational stress had a direct negative effect, and resilience and professional benefits partially mediated the impact of stress on work-related wellbeing.

Family doctors’ presenteeism (Yu et al.) was negatively correlated with quality of work life and professional identity and positively correlated with job burnout. Hierarchical linear regression revealed significant impacts of work stress, professional behavior tendency, professional values, professional belonging sense, emotional exhaustion, depersonalization, and professional efficacy on presenteeism. Among medical students in the post-pandemic era (Zhong et al.), future work self-salience positively predicted occupational sense of mission, with professional identity partially mediating this relationship. Latent profile analysis identified three distinct profiles—high future work self-salience/high professional identity, moderate/moderate, and low/low—with the high/high profile exhibiting the highest occupational sense of mission.

Young medical professionals’ professional identity was examined within the New Quality Productive Forces framework (Zhang et al.), with perceived social support significantly influencing professional identity (total effect b=0.625), comprising direct effect b=0.374 (59.80%) and indirect effect mediated by role cognition b=0.251 (40.20%).

A conceptual analysis of new nurse workplace adaptation using Walker and Avant’s method (Xu et al.) identified three attributes: continuous dynamic adjustment, achievement of internal-external equilibrium, and integration into team collaboration. The analysis introduced the concept of “sacrificial adaptation”—a state where nurses compromise internal wellbeing for superficial integration—exposing a fundamental flaw in management strategies that prioritize behavioral compliance over authentic wellbeing. The provision of medication-free services within traditional mental health wards (Beyene et al.) was explored through qualitative focus groups, revealing an overall theme that medication-free services clash with a traditional medical system due to fundamental differences in philosophy, structure, power dynamics, and focus. Systemic changes were identified as necessary to create a more flexible, empowering, and relationship-focused milieu.

Moral dilemmas during the COVID-19 pandemic (Vecchio and Colombo) were examined from an organizational perspective, exploring how organizational structures, policies, and leadership influenced healthcare professionals’ ethical decisions through processes of sensemaking and ethical suffering. A cross-sectional study of 8,071 physicians in Gansu Province’s infectious disease sentinel institutions (Li et al.) found that coping style mediated the relationship between job satisfaction and burnout, while neurotic personality moderated these relationships, providing insights for targeted organizational interventions.

## Conclusion and future directions

Collectively, the 33 articles in this Research Topic paint a comprehensive picture of the mental health challenges facing healthcare professionals and the multifaceted strategies needed to address them. Several cross-cutting themes emerge. First, the burden is substantial and pervasive—from the 70.48% burnout prevalence among Chinese residency trainees (3) to the 51.5% anxiety among Palestinian healthcare workers (4)—yet it varies significantly across settings, roles, and contexts, demanding tailored rather than one-size-fits-all approaches. Second, protective factors operate at multiple levels: individual (resilience, coping strategies, mentally active behaviors), interpersonal (social support, peer relationships), and organizational (supportive supervision, work-life balance, institutional trust). Third, effective interventions span a spectrum from low-tech (peer support, narrative nursing, meaning-making) to high-tech (digital mental health, biofeedback), suggesting that diverse tools are needed to meet diverse needs. Fourth, the voices and lived experiences of healthcare professionals themselves must inform the design and implementation of support strategies—as demonstrated by the group concept mapping approach that enabled ICU professionals to define their own coping priorities (Olander et al.).

As we look forward, several priorities warrant attention. Longitudinal research is needed to track how mental health trajectories evolve and how interventions exert their effects over time. Implementation science approaches can help bridge the gap between evidence and practice, particularly in resource-constrained settings—as highlighted by the implementation gap between high importance and low use of supportive professional practices (Olander et al.). Health-promoting behaviors (5) and mentally active sedentary behaviors (He et al.) represent accessible, low-cost protective factors that can be integrated into daily routines. The concept of “sacrificial adaptation”(Xu et al.) reminds us that genuine wellbeing cannot be achieved through superficial compliance alone.

Ultimately, promoting mental health among healthcare professionals requires systemic change—not merely individual resilience training, but fundamental transformation of working conditions, staffing models, and organizational cultures that prioritize the wellbeing of those who care for us all. Special attention is called for comprehensive support systems, national policies, and cross-cultural research to improve healthcare workers’ mental wellbeing (6). Addressing both contextual and psychological factors is critical for reducing burnout and promoting sustainable professional development across healthcare settings worldwide.

We thank all authors who contributed their scholarship to this Research Topic and hope this collection serves as a valuable resource for researchers, clinicians, policymakers, and advocates committed to this essential mission.
